# The Clinical Society of Maryland

**Published:** 1888-01

**Authors:** 


### Society Reports.

### THE CLINICAL SOCIETY OF MARYLAND.

STATED MEETING HELD OCT. 21, 1887.

The 19th meeting of the Clinical Society was called to order by the President, Dr. N. G. Keirle in the chair.

### EPHITHELIOMA OF THE SCALP.

Dr. W. H. Norris related a case of supposed epithelioma of the scalp and exhibited the patient. He was a colored man, æt. 46 years, occupation brick-maker. His family history was not well known. So far as could be obtained there was no hereditary taint. He had a sore on his penis when quite young. When a boy he received a severe burn on the scalp. Last March a pimple made its appearance on his scalp and this ulcer resulted from it. Four months ago the ulcer was one-third the size it is to-night. The results of treatment have been negative. At first he tried a concentrated solution of alum and sugar of lead with some good effects. Next ferri. per sulph. was used with no good results; then iodoform and lastly permanganate of potassium. He believes the trouble to be an epithelioma, though this disease is rare in the African race, and its rapid growth, too, would be in favor of its not being an epithelioma.

Dr. J. W. Chambers then showed a specimen of

### CYSTIC SARCOMA OF THE INFERIOR MAXILLARY

and the patient from whom it was taken. Patient, colored, male, æt. 42 years, laborer; family history good. He entered the City Hospital on the 12th day of September with an enlargement on the right lower jaw; which extended from the last incisor tooth to the right condyle and involved nearly all this side of the face. It extended inwards and displaced the tongue so as to interfere decidedly with mastication. The growth was cystic in character. It began sixteeen years ago after the removal of a tooth and at first grew slowly. Two years ago, though, it began to grow more rapidly. There was no involvement of the skin nor the neighboring glands. On the 16th day of September an incision was made and the tumor dissected out mostly with Paquelin's cautery. There was not much hemorrhage. The inferior maxillary was then sawed off and disarticulated. The wound was dressed with hy. bichlor. and iodoform. On the sixth day the stitches were removed. There was no shock. The temperature never rose above 99 degrees. His speech is now much better. Since the operation the patient has developed some mental derangement. The results show what can sometimes be accomplished even when the conditions presented are apparently hopeless.

DISCUSSION.

Dr. O. J. Coskery thinks that Dr. Chambers should be congratulated on the good results gotten in this case. He then showed a specimen and related a case of keloid of unusual size. The patient was a colored man, æt. 25 years. The tumor extended from the lobe of the ear to the clavicle. In 1884 while being shaved a bump was cut and a sore resulted from it. He applied nitric acid to it which cooled it, he said, but afterwards the pimple grew rapidly. On October 18, 1887, he removed the growth. An incision was made from the lobe of the ear to the clavicle, and it was about six inches long. When the tumor was removed it left a gap two inches wide. The edges were then brought together. There still remains a gap about five inches long and one-half inch wide. Patient never had temperature above 99 degrees. Hy. bichlor. dressing was used. He exhibited the tumor on account of its size, as it was the largest keloid he had ever seen.

Dr. Wm. Rickert related a case of

OSTEOMA OF THE INFERIOR MAXILLARY

and exhibited both the patient and specimen. Patient was colored, male, about 21 years of age. He had been troubled with his jaw for about four years and under treatment for one year. He came under his care about three months ago, and finally became so much affected that he could hardly get the tip end of a lead pencil between his teeth. He operated then, and made a partial excision of the inferior maxillary on the right side.

Dr. R. Winslow showed a specimen of

PERI-OSTEO-SARCOMA.

from the lower jaw; patient was an Irishman, æ. 67 years. He had the growth for twelve months before it was removed. The man did well for awhile, but now has a lump on the tissue outside, probably an inflammation. This boy, (Dr. Rickert's case) came to him sometime ago with a lump in the jaw and he excised a wedge shaped piece according to Esmarck and afterwards he could open his mouth as well as now. This condition is osteitis and is simply inflamed bone, which increases in size and prevents him from opening his jaws.

Dr. W. H. Norris said that he wanted it decided in his case whether the trouble was malignant or not, and what would be the treatment.

Dr. I. E. Atkinson said that there was no doubt in his mind about the diagnosis of epithelioma being correct. Ulcerations of that character are usually either syphilis or epithelioma. The best thing to be done is to leave it alone. Under stimulating ointments, etc., new growth will take place more frequently. Most probably the growth has involved the bone. Referring to Dr. Coskery's case he thought that those conditions which are so common in the colored race are not true keloid, but false. True keloid is rather rare. It begins as an oat-shaped growth and is usually situated about the breast. The false very frequently begin after burns. Keloids soften up after a time. The ordinary form recurs frequently, and occasionally they disappear as years go by. The true form in the colored race, he has never seen occur after slight wounds.

Dr. L. McLane Tiffany showed several photographs of

KELOID GROWTHS.

He spoke of the largest one and said that it weighed forty-two ounces. He thinks all of them are larger than the one shown by

Dr. Coskery. He did not recognize the difference between true and false keloid as referred to by Dr. Atkinson. They are more common in the colored race, and especially in the young colored race. He had seen them softer in the older colored people. When seen in the young person, non-interference until later in life, then an operation will meet with good results. After a patient passes beyond thirty years of age, they are not apt to recur. There may be an hypertrophic scar, but no lump. Two cases he spoke of gave no history of injury.

Dr. Hiram Woods had seen several cases of keloid at the Presbyterian Eye and Ear Hospital, only one of which occured in the white, the rest in negroes. The oldest patient was 42 years of age and had dumb-bell lumps on her ears, which had become somewhat soft. Ear authorities say that when we operate we must try and get the wound to heal by first intention. He once operated and did not bring the edges close together and the growth recurred. The more closely we can get a wound to heal by first intention the better will be the results.

Dr. R. Winslow said that as these tumors sometimes develop from a puncture with a point of a pin, he did not see why they would not sometimes recur even if the edges are brought close together.

Dr. Rickert thinks that the softening is more common in the white race, and when they are removed will not return so commonly as in the negro.

Dr. I. E. Atkinson said that his experience was based entirely upon observations in the colored race, He said that Dr. A. B. Arnold once reported a case where a keloid disappeared under the use of collodion. The keloid that he had removed in the white did recur.

Dr. Hiram Woods said that he knew that keloids did develop from the puncture of a pin point, and he simply wanted to say, that if, after operating, we can reduce granulation to the minimum, there will be a better chance to get over the disease.

Dr. L. McLane Tiffany, said that it was one of the rarest of jaw tumors. There are only a few reported. Evidently they are cysts lined with epithelium, very much like an ovarian cyst, but it is not settled yet as to what they are.

Dr. J. T. Branham said that he had seen a keloid in an old colored man. It was flat, situated on the breast and ulcerated, which he says is rather common. There is no scar tissue when wounds heal by first intention. The case of Dr. Rickert had changed somewhat since he was under Dr. Winslow's charge. The tumor is most probably the result of an inflammatous process and probably not malignant. The removal of which he thought was the best thing to do in order to allow the patient to eat and talk comfortably.

Dr. Rickert said that his only object was to give this comfort, and he believed it has done vast good to the patient.

Dr. Chambers said that he had spoken very little about the finer probabilities in his case. He classed it as cystic sarcoma. There was no enlargement of the glands, and the growth was slow. The tumor, when removed, weighed about four or five pounds.

Dr. Keirle said that these growths are very similar to an ovarian cyst. The larger cyst contained chocolate-colored fluid, and the smaller one another colored fluid. They are lined with epithelium like an ovarian tumor.

Dr. Gorgas then read a paper entitled "A Case of Puerperal Septicæmia. The patient was a German woman, æt. thirty years, and the mother of four children. She was delivered on Sept. 12th, of triplets. Six days after delivery she was taken with a severe chill, which was followed by high fever, profuse perspiration, cessation of the lochia, nausea, loss of appetite and strength. Her temperature ranged from 102 degrees in the morning to 106 degrees in the evening for six days. Under the use of intra-uterine injections of warm carbolized water and the administration of anti-pyretic remedies viz.: quinine and muriate of quininia and urea., with stimulants and good food, the patient recovered , and three weeks from the date of her confinement she was able to leave her bed, and is now attending to her household duties.

